# Engaging new partners in human papillomavirus (HPV) vaccine promotion: Considerations for training private practice dental professionals in HPV vaccine promotion

**DOI:** 10.1111/jphd.12566

**Published:** 2023-03-09

**Authors:** Natoshia Askelson, Grace Ryan, Susan McKernan, Aaron Scherer, Eliza Daly, Lejla Avdic

**Affiliations:** 1 Department of Community and Behavioral Health, College of Public Health, University of Iowa, Iowa City, Iowa, USA; 2 Department of Preventive and Community Dentistry, College of Dentistry, University of Iowa, Iowa City, Iowa, USA; 3 Department of Internal Medicine, Carver College of Medicine, University of Iowa, Iowa City, Iowa, USA

**Keywords:** continuing education program, dental hygienists, dentists, human papillomavirus, surveys

## Abstract

**Objectives:**

With rates of the human papillomavirus (HPV) vaccination remaining low and rates of oropharyngeal cancer rising, engaging new partners to promote vaccination is necessary. We aimed to identify dental hygienists’ and dentists’ knowledge about HPV, the HPV vaccine, and preferences for continuing education.

**Methods:**

This mixed-methods study recruited dental hygienists and dentists working in private practice in Iowa to participate in a mailed cross-sectional survey (dental hygienists) and qualitative telephone interviews (dental hygienists and dentists). Survey and interview topics included existing knowledge about HPV vaccination, HPV vaccination promotion efforts, barriers to HPV vaccine promotion, and preferences for continuing education (CE).

**Results:**

We received 470 surveys from dental hygienists (response rate = 22.6%) and interviewed 19 dental hygienists and 20 dentists. Vaccine efficacy and safety, along with communication strategies, were key topics of interest for CE. Dental hygienists’ most commonly identified barriers were lack of knowledge (67%) and low comfort levels (42%).

**Conclusions:**

Knowledge was identified as a key barrier to providing a strong recommendation for HPV vaccination and convenience was the most important factor to consider for any future CE. Our team is in the process of designing a CE course based on this information to help dental professionals effectively engage in HPV vaccine promotion in their practices.

## INTRODUCTION

The American Academy of Pediatric Dentistry and the American Dental Association encourage dental providers to educate parents on the relationship between the human papillomavirus (HPV) and oropharyngeal cancers and to counsel patients for vaccination according to CDC recommendations [1, 2]. HPV causes an estimated 44,000 cases of cancer each year with 19,000 of those being cancer of the oropharynx [3]. A highly effective vaccine is recommended for adolescents aged 11–12 years old (the series may be started as early as age 9) to protect against future HPV infection and its associated cancers [4]. Recently, the Food and Drug Administration expanded its approval of the HPV vaccine to include the prevention of HPV-associated head and neck cancers [5], lending further support for including dental providers in efforts to promote HPV vaccination and the prevention of HPV-associated oral cancers.

Despite the available vaccine, widespread educational efforts [6], and evidence-based interventions (EBIs) to improve vaccine promotion in medical clinics [7, 8], vaccination rates are still below the Healthy People 2030 goal of 80% completion for adolescents. According to the 2019 National Immunization Survey-Teen, only 54% of adolescents aged 13–17 were up-to-date on HPV vaccination [9]. HPV-associated oropharyngeal cancer (OPC) cases have risen in recent years, now reaching an estimated 13,500 cases per year compared to the ~10,900 HPV-associated cases of cervical cancer per year [3]. A majority of the HPV-associated OPC cancer burden falls on men, who account for ~82% of cases [10]. These low rates of vaccination coupled with increasing incidence of HPV-related cancers suggest that increased efforts to promote vaccination are needed. Engaging new partners, like dental providers, could be an effective way to promote vaccinations.

Dental hygienists and dentists routinely provide preventive care to adults involving tobacco cessation counseling [11] and dietary and nutrition counseling [12]. Additionally, dental hygienists and dentists provide oral cancer screenings and risk assessment as a standard of care [1]. Expanding their preventive services for adolescent patients to include HPV vaccine promotion and referrals to appropriate clinics represents a natural extension of existing health promotion activities. Additionally, with the current recommendation for children to visit a dentist twice a year versus the recommendation to have a well-child visit once a year, adolescents may see a dental provider more frequently than a health care provider [13]. Thus, some adolescents may be more likely to see a dentist than a physician in a given year, offering additional opportunities for healthcare providers to discuss the HPV vaccine at appropriate ages.

Parents have shown positive perceptions of dentists and dental hygienists promoting the HPV vaccine and providing HPV education during dental visits [14, 15]. Past work has assessed dental providers’ views on HPV and oral cancer control, with findings suggesting that dental providers could be successful agents in promoting the HPV vaccine [16–19]. However, dental providers have identified a need for professional education focused on HPV and OPC to overcome their perceived lack of knowledge related to these topics and support them in engaging in HPV vaccine promotion efforts [19, 20].

In order to develop effective interventions that encourage dental offices to become engaged in HPV vaccine promotion, further research needs to be done on providers’ current knowledge, barriers, and training needs. The study presented here highlights findings from a mixed-methods project drawing on the perspectives of both dentists and dental hygienists working in private practice to design continuing education (CE) opportunities that address knowledge gaps. The results of this study will be used to inform the development of future interventions targeting private practice dental offices.

## METHODS

We designed a mixed-methods study to explore existing HPV vaccine promotion efforts among dental hygienists and dentists practicing in Iowa, as well as their willingness to participate in continuing education programming focused on the HPV vaccine. All data collections were completed between May and August of 2019 by MPH-level graduate research assistants. Our study was designated nonhuman subjects research by the University of Iowa Institutional Review Board.

### Mail survey: Dental hygienists

Utilizing existing research [16, 21, 22, 23], we designed a 31-item survey to assess individual characteristics and behavior, perceived barriers that would make it difficult to educate parents of adolescent patients about HPV vaccination (13 barrier options included), willingness to participate in CE about HPV, the vaccine, and oropharyngeal cancer (OPC) (1 question), prior CE taken on HPV vaccination and OPC, and topics to be covered in a CE course (5 potential topics included). Our survey instrument was reviewed for face validity and pre-tested with a group of dentists and dental hygienists (*n* = 4). We used a list of licensed dental hygienists provided by the Iowa Dental Board and mailed surveys to 2074 hygienists with pre-paid return envelopes. Our research team sent two follow-up mailings between May and June 2019: a postcard reminder 10 days after the initial survey and a second survey at 3 weeks following the initial mailing. Additional details on the survey and results are detailed elsewhere [20]. To analyze survey data, frequencies and descriptive statistics for all variables were generated using IBM SPSS Statistics 23.

### Telephone interviews: Dental hygienists and dentists

We conducted telephone interviews with dental hygienists focusing on HPV promotion efforts in their practices, interest in and preferences for continuing education, and any logistical barriers to CE participation. In Iowa and many other states, dental hygienists are required to work under the supervision of a licensed dentist. Therefore, parallel interviews of dentists were conducted to provide perspectives regarding HPV vaccine promotion and preferences around potential CE courses on this topic. Qualitative data were elicited using an interview format and process that has been described previously by Polkinghorne [24].

### Dental hygienist interviews

For the dental hygienist interviews, we recruited from the list of licensed dental hygienists. Using the zip code provided, we split potential participants by congressional district (*n* = 4) and randomly selected 25 hygienists from each. We did two waves of recruitment for a total of 200 mailed invitations. After the initial recruitment letter, we sent a follow-up postcard to all potential participants. To complement the recruitment methods described above, we also conducted in-person recruitment at two local dental professional conferences.

### Dentist interviews

We obtained access to the University of Iowa College of Dentistry Alumni Directory (*n* = 1642) and eliminated respondents without an email address which resulted in 905 potential participants. We first drew a random sample of 300 potential participants and conducted initial outreach via email and followed up via phone. When this did not result in a satisfactory number of interviews, we sent recruitment materials via email to the rest of the sample (*n* = 605).

All dentists and dental hygienists who completed an interview were mailed a $25 gift card.

### Data analysis: Interviews

Interviews were audio recorded and then transcribed by a third-party service. Our team reviewed interview transcripts to ensure thematic saturation was reached. We deductively developed codebooks for each set of interviews and had two research assistants independently code the same two transcripts using NVivo. Then, these research assistants met with a third member of the research team to discuss discrepancies in coding, establish concordance between coders, and revise the codebook [25]. The remaining transcripts were divided between the two research assistants to complete coding.

## RESULTS

### Participants

We received 597 surveys from dental hygienists (30.4% response rate). Since dental hygienists and dentists working in private practice were the focus of the current analyses, we eliminated 127 respondents who worked in dental settings other than private practice (e.g., community health centers or other public health settings), where scope of practice and required levels of supervision can vary. This resulted in a response rate of 22.6%. Our team completed interviews with a total of 23 dental hygienists and 30 dentists. Here, we report the results from 19 dental hygienists and 20 dentists working in private practice. Demographic information for participants in both the survey and interviews is presented in Table 1.

### Barriers to HPV vaccine promotion

To understand barriers to discussing HPV and the link with oropharyngeal cancer, our survey asked dental hygienists what inhibits them from discussing HPV’s link with OPC. Only one-fifth of them (22.3%) reported that they would have no difficulty talking about the HPV vaccine with parents of their adolescent patients. The most commonly cited barriers were:

- not knowing enough about the vaccine (67.0%),
- not feeling comfortable talking to parents about the vaccine (41.9%), or
- believing that vaccines are not in hygienists’ scope of practice (31.5%).

Very few dental hygienists reported believing that the vaccine was not safe (9.8%) or efficacious (1.5%). Moreover, few respondents identified that their religious/spiritual (2.3%) or political beliefs (0.4%) would prevent them from discussing the vaccine. Frequencies for all barriers considered by the survey of dental hygienists are presented in Table 2.

One of the main barriers cited among both dentists and hygienists in their telephone interviews was lack of knowledge about the vaccine (Table 3). In addition to lack of knowledge, some dental hygienists also noted that there were potential structural barriers within their dental offices. These structural barriers included limited time and open-office settings that could potentially make conversations about HPV with patients and their parents more challenging.

### Continuing education preferences

Three survey items gauged the level of previous participation in continuing education on HPV vaccination and OPC among dental hygienists. While the majority (88.5%) reported completing a course on oropharyngeal cancer, few had completed courses on HPV (25.6%) or HPV vaccination (11.2%). However, 87.2% were willing to complete a CE course in the future on the topic of HPV and oropharyngeal cancer. Only 6% of respondents reported that they did not want any CE on HPV and oropharyngeal cancer.

We asked about what topics they would be interested in and the best format to deliver them. Topics of highest interest were:

- Vaccine efficacy (89.4%)
- Vaccine safety (82.5%)
- Communication strategies to speak with parents (74.5%)

Lunch and learn or in-person meetings were the most popular formats for a CE class with 61.2% and 60.4%, respectively identifying a preference for these. Slightly more than half of dental hygienists surveyed (52.9%) reported wanting webinars and a minority (20.2%) said that workshops would be preferable.

Table 3 highlights relevant themes and quotes from interview data on previous CE experiences and preferences for future CE. In interviews, dentists and dental hygienists reported that they were willing to learn more about HPV and the HPV vaccine and potentially to incorporate promotion strategies in their office practice. Interview data showed that dental hygienists in our sample have a strong interest in continuing education and training on how to recommend the vaccine. In fact, many hygienists reported that they believed that, with training, talking about the HPV vaccine with parents would be easy to incorporate into their work.

Interviewed dental hygienists were asked to share some of topics they would prefer future CE courses to include. They requested education on general statistics related to HPV and its connection to oral health, as well as effective communication techniques. Dentists also voiced support for CE formats that would provide general information on HPV and its connection to oropharyngeal cancer. Overall, dentists and dental hygienists appeared to be open to different formats for CE. However, many dentists noted that the most important factor for an appealing CE course was convenience.

## DISCUSSION

With the recent approval of the HPV vaccine as oropharyngeal cancer prevention our study highlights an impor tant area of opportunity for dental hygienists and dentists. Our results confirm that this is a group that is willing to participate in continuing education so that they can better protect their patients and promote the HPV vaccine. Although a majority of dental hygienists in our study were willing to recommend the HPV vaccine to parents of their adolescent patients, very few reported having received any CE that specifically addressed HPV or the HPV vaccine. The primary barriers reported by dental hygienists and dentists in our study were lack of knowledge about the HPV vaccine and being uncomfortable educating parents about it. Future planners of CE need to consider the perspectives of dentists and dental hygienists, and the barriers they see to engaging in this type of work, to tailor and implement effective educational programming.

While the dental hygienists and dentists in our study did not express confidence in their ability to talk to parents, previous research has found that parents would trust dentists’ and dental hygienists’ qualifications to provide HPV vaccine counseling and they would expect their dental providers to talk to them about HPV [15]. This finding of a lack of confidence is also similar to what has been observed in other studies [26, 27]. With evidence that parents would be accepting of this kind of information coming from dental hygienists and dentists, CE efforts can focus on eliminating barriers perceived by dental hygienists and dentists. Previous research has documented and identified knowledge deficits among dental providers related to HPV and oropharyngeal cancer [16, 18], and also supports associations between knowledge and the likelihood that providers’ will talk to their patients about HPV and the linkage with oral cancer [18]. Our results echo these findings that there is a lack of knowledge among dentists and dental hygienists about HPV and the vaccine [28] and expand on this research by identifying specific topics and formats for continuing education.

Notably, one barrier identified by both dentists and dental hygienists in this study was how structural design of the office (i.e., having open office spaces) adversely affect their comfort speaking with parents about HPV vaccination—a potentially sensitive topic for parents and providers. Other studies have also noted that the open space of dental offices and potentially sensitive nature of this topic would be a barrier to discussing HPV vaccination with parents and adolescents [29, 30]. Open operatories are perceived to limit privacy and may inhibit conversation. Future investigations should explore whether a change in office workflow may be necessary to build in opportunities for HPV vaccine promotion (e.g., a private patient room), or whether masking noise in the form of water sounds, music, or white noise machines would be beneficial [31]. If found to be beneficial, strategies about how to eliminate these barriers related to the open office setting could also be included in CE courses.

In terms of specifics for what should be covered for CE and how to best deliver it, dental hygienists and dentists in our study offered important insights. Although few dental hygienists expressed concerns over safety of efficacy of the vaccine, these were key issues that they wanted to be covered in CE. This discrepancy may indicate a lack of confidence around hygienists’ skills at communicating with parents on these topics and feeling that they have all the facts to share with parents. One strategy could be to explore existing CE courses that focus on these topics and expand on these offerings to meet the needs of dental professionals [32]. Regarding delivery of CE content participants preferred lunch and learns or in-person meetings, and over half of dental hygienists (52.9%) indicated a preference for webinars. For states, like Iowa, where this was conducted, webinar-based training would also provide the opportunity to reach more geographically disparate practices efficiently. Moreover, given restrictions due to COVID-19, webinars may be the best option for CE delivery. Our study participants also emphasized the importance of convenience, in terms of time required for the training and logistics, in receiving the information.

Finally, while knowledge gaps exist for both dentists and dental hygienists, as shown in this study, there is evidence of the particular importance of focusing CE efforts for dental hygienists. Previous research identified dental hygienists as potentially being more effective educators and disseminators of this kind of information due to their longstanding relationships with parents [20]. Therefore, while all dental professionals should have access to CE opportunities to provide education on HPV vaccination and HPV-related cancers, it will be particularly important for dental hygienists.

## STUDY STRENGTHS

We developed the data collection instruments by adapting items from validated surveys and developing new items based on findings from previous research. Findings from the current study are immediately actionable and highlight areas related to knowledge and self-efficacy that should be addressed in order to empower dental hygienists and dentists as effective HPV vaccine promoters. Finally, our study is strengthened by our use of a mixed-methods approach and our triangulation of survey data from hygienists with interview data from both dentists and dental hygienists.

## STUDY LIMITATIONS

The primary limitation to our research is that both our quantitative and qualitative data may have been affected by response bias. While our original target population captured nearly the entire dental provider population in the state, it is possible that those who answered our survey or participated in an interview already believed that HPV vaccination was an important topic to address in dental settings and therefore non-responders may be more unwilling to engage in these types of efforts. Additionally, for interviews with dentists we relied on the alumni list from the University of Iowa. With over 76% of private practice dentists in Iowa attended University of Iowa, we were not able to reach all dentist practicing in the state, but this list has contact information for the majority.

## CONCLUSIONS

Key findings from this study can be used to design specific strategies to improve dental hygienists’ skills and confidence in their ability to advocate for HPV vaccination recommendation to parents of adolescent patients. It is important to develop CE courses that increase the dental hygienists’ confidence in educating patients about this healthcare issue. It will be important to use CE courses as opportunities to also directly address barriers identified by dental hygienists and empower them to feel confident in having these conversations. This team’s next steps are to pilot an intervention with a CE component to train dental hygienists in effectively recommending the HPV vaccine to parents of their adolescent patients. The ultimate goal of providing tailored and targeted CE to dental professionals is to engage new partners to promote HPV vaccination for the adolescent population to prevent future HPV-related cancers.

## Figures and Tables

**TABLE 1:** Demographic characteristics of dental hygienists and dentists participating in survey and interviews.

	Dental hygienists				Dentists Interviews (*n* = 20)	
	Survey respondents (*n* = 470)		Interviews (*n* = 19)			

	*n* (%)	Mean (SD)	*n* (%)	Mean (SD)	*n* (%)	Mean (SD)
**Sex**						
Male	–	–	0 (0)	–	12 (60.0)	–
Female	–	–	19 (100)	–	8 (40.0)	–
**Age (years)**						
18–26	14 (3.0)	–	0 (0)	–	1 (5.0)	–
27–39	176 (37.4)	–	3 (15.8)	–	8 (40.0)	–
40–59	228 (48.5)	–	12 (63.2)	–	3 (15.0)	–
60 +	52 (11.1)	–	4 (21)	–	8 (40.0)	–
**Education and certification**						
Hygiene certificate	213 (45.3)	–	2 (10.5)	–	–	–
Associate’s degree	316 (67.2)	–	11 (57.9)	–	–	–
Bachelor’s degree	147 (31.3)	–	6 (31.6)	–	–	–
Masters’ degree or higher	5 (1.1)	–	0 (0)	–	–	–
**Adolescents as a percentage of all patients (%)**	–	23.9 (12.3)	–	23.2 (18.9)	–	20.3 (9.9)

**TABLE 2:** Dental hygienists’ perceived barriers in discussing human papillomavirus (HPV) vaccination with parents of adolescent patients, 2019 Survey of Iowa Dental Hygienists (*n* = 470).

Barrier^a^	*n*	%
I do not know enough about the HPV vaccine	315	67.0
I do not feel comfortable talking to parents about HPV vaccine	197	41.9
Vaccines are not within my scope of practice	148	31.5
Open office setting	146	31.1
The office does not have the privacy needed for these conversations	132	28.1
Not enough time	114	24.3
I would not have any difficulty talking about the HPV vaccine with parents	105	22.3
I do not think the HPV vaccine is safe	46	9.8
My religious or spiritual beliefs	11	2.3
I do not think the HPV vaccine prevents cancer	7	1.5
Vaccines are not relevant to oral health	6	1.3
I do not think HPV vaccine is effective at preventing HPV-associated oral cancers	6	1.3
My political beliefs	2	0.4

a Respondents were asked to check all barriers that applied.

**TABLE 3:** Summary of human papillomavirus (HPV) and HPV vaccine themes, sub-themes, and representative quotes from interviews of dental hygienists and dentists in private practice.

Theme	Sub-theme	Representative quotes
Personal Barriers to Vaccine promotion	Lack of information	• “I feel like I just don’t have enough background information, enough solid information to feel comfortable with talking to parents about that” (hygienst_4.5.2019) • “I’ll be honest, I do not know much about HPV vaccination or what is the recommendation for it. I will say, I honestly don’t know that much about it.” (dentist_8.8.2019)
Structural barriers to vaccine promotion	Time	• “Probably just time, if I bring the vaccine up, I’d have to spend more time talking about it. I can’t just say, ‘Hey, go get this vaccine’, without giving some background. So again, it would take up time” (hygienist_5.3.2019)
	Open-office design	• “Just to be able to talk openly and with the parent and the adolescent, our hygiene rooms and all of our operatories are pretty open to each other and there just would not be a good setting for doing that without other people being able to hear.” (hygienist_7.16.2019)
Willingness to promote HPV vaccine	Importance of professional training	• “You know, if I felt like I really had the facts on it, yeah, I think I wouldn’t, yeah. It would be easy talking to them about it.” (hygienist_5.3.2019) • “At first it might be difficult but I would think with the proper training that it would become easier the more you did it.” (hygienist_7.12.2019) • “Like I said, I just need to be a little bit more educated on it, so that I could become more confident about it. That’s where I’m falling right now. It’s a confidence thing. I need to become educated, because they’re going to have questions, and I want to have answers for them when they do have questions.” (hygienist_5.13.2019) • “Well, I mean, if I was educated on what the connection was, then I would feel very likely to be able to discuss that with the child and the parent.” (hygienist_4.2.2019)
Preferred CE Topics and format	Hygienists: Preferred topics to be covered in CE	• “Probably… a little more about HPV in general. I know there’s all these strains of the human papilloma virus, but maybe a little more training on that whole topic of the human papilloma virus so that… I mean I think I know some, but I probably don’t know as much as I need to and have them really stress why it’s important and how we need to go about bringing this conversation up and start making people more aware.” (hygienist_7.1.2019) • “Just the facts, so they can prevent, just easier, best time to get the vaccine. Yeah, just kind of the basic facts.” (hygienist_5.2.2019) • “Just hardcore stats. Just the basic stuff that people are gonna wanna know, and how it can effect a patient, especially with cancer, and just the basic information that people can understand, and connect the dots, because if it’s too wordy, and it’s too over your head for the basic person, has no knowledge of cancer or dental stuff, you don’t want to hand out something, or tell them something that they’re just not gonna understand.” (hygienist_3.29.2019)
	Dentist: preferred format for CE	• “I think presentations, like visual presentations with background information, statistics on HPV, the link between oropharyngeal cancer and HPV, and just having a lot of education topics that reinforce why we’re promoting the HPV vaccine. I think it can be in a classroom setting. And I think also having copies of the information, like written out statistics and the presentation would be helpful to kind of remind them of what was talked about in the presentation.” (dentist_8.3.2019)
	Importance of convenience for CE	• “The logistics would be the determining factor on that, but very willing if it was reasonable and convenient” (dentist_7.27.2019) • “If it was convenient, easy to get to, like at our local community college, or a hotel, or conference center, or whatever, I’m sure we could make that happen.” (dentist_7.27.2019) • “I’m not as fond of self-paced, go online, learn on your own, because I think it’s helpful to have interaction with the people and questions. Does that make sense?” (dentist_8.16.2019)
